# Large Nonfunctioning Anterior Mediastinal Epiaortic Paraganglioma

**DOI:** 10.1155/cric/4216128

**Published:** 2026-03-11

**Authors:** Batoul Abbas, Aya Alhelou, Eman Darrh, Batool Marwa, Albaraa Bara, Mohammad Bashar Izzat

**Affiliations:** ^1^ Department of Surgery, Damascus University Faculty of Medicine, Damascus, Syria, damascusuniversity.edu.sy

**Keywords:** case report, mediastinum, neoplasm, paraganglioma, pathology, surgery

## Abstract

We present a very rare case of a carotid body tumor accompanied by a large nonfunctioning epiaortic paraganglioma requiring ascending aorta replacement. Even though multiple paragangliomas in different locations in a single patient are highly uncommon, preoperative discovery of an extrathoracic neuroendocrine tumor should prompt full screening for the presence of multiple tumors.

## 1. Introduction

Paragangliomas are rare neuroendocrine tumors arising from chromaffin cells along the paravertebral and para‐aortic axes [1, 2]. The posterior mediastinum is the most frequent site for intrathoracic paragangliomas, whereas anterior mediastinal paragangliomas are exceedingly rare [3]. Moreover, multiple paragangliomas in different locations in a single patient are highly uncommon.

We present a very rare case where a carotid body tumor was accompanied by a large nonfunctioning epiaortic paraganglioma requiring ascending aorta replacement.

## 2. Case Report

A 54‐year‐old male presented to our hospital with recurrent severe chest pain at rest accompanied by dyspnea. He had a 5‐year history of well‐controlled hypertension. Physical examination, ECG, laboratory tests, and chest x‐ray were all within normal limits. Transthoracic echocardiography did not reveal any abnormal findings and confirmed good left ventricular systolic function with an ejection fraction of 60%. Urgent cardiac catheterization showed atherosclerotic coronary vasculature, with total occlusion of the left anterior descending (LAD) artery beyond its origin and a 70% stenosis of the obtuse marginal artery. An attempt to perform percutaneous transcatheter intervention to reopen the LAD was not successful; hence, the patient was referred for surgical coronary revascularization. Preoperative preparation included a carotid duplex scan that did not detect any extracranial vascular abnormality, but a highly vascularized 20 × 17 mm mass was identified on the right side of the neck, located between the external and internal carotid arteries, consistent with a carotid body tumor.

Surgical intervention was carried out under general anesthesia. A full median sternotomy was performed, and the skeletonized left internal mammary artery (LIMA) was harvested. Upon opening the pericardium, a 40 × 50‐mm firm, dark red, and highly vascularized mass with an extensive venous mesh was discovered covering the anterior aspect of the proximal ascending aorta (Figure 1a). Cardiopulmonary bypass perfusion was established using distal aortic and right atrial cannulas, the aortic cross‐clamp was applied at the distal extent of the ascending aorta, and myocardial protection was achieved using antegrade cold blood del Nido cardioplegia.

Figure 1(a) Operative view of the epiaortic mass covering the anterior aspect of the proximal ascending aorta and (b) ascending aorta replacement with coronary artery bypass grafts.

**Figure:**
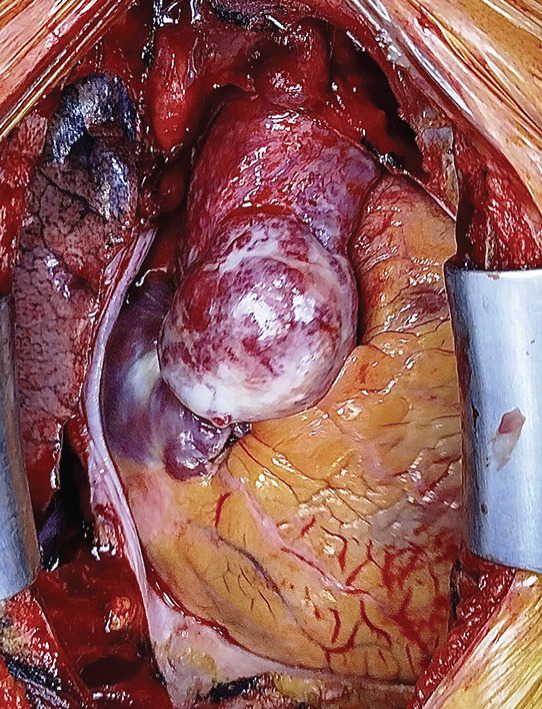
(a)

**Figure:**
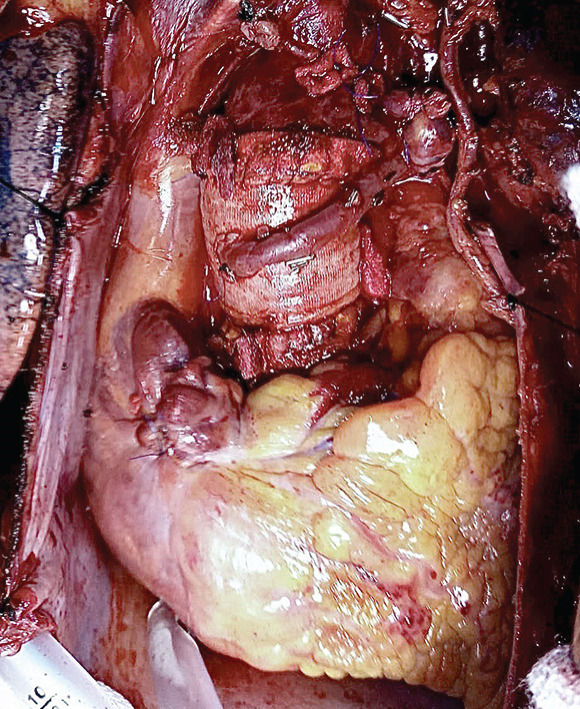
(b)

The ascending aorta was incised transversely 10 mm above the mass. The latter was found to be firmly adherent to the aorta without invading the aortic wall. A second transverse incision was made 10 mm below the mass at the level of the aortic commissures to inspect the aortic cusps, which were intact. The ascending aorta was excised together with the mass and was replaced with a Dacron tube graft using continuous 4‐0 polypropylene sutures. Finally, the LIMA was used to revascularize the LAD, and a saphenous vein graft was used to revascularize the obtuse marginal artery. The proximal end of the saphenous vein graft was anastomosed to the anterior aspect of the Dacron tube graft (Figure 1b). The patient was weaned off cardiopulmonary bypass easily without inotropic support, and the postoperative course was uneventful. The patient was discharged from the ICU 24 h after surgery and from the hospital after 6 days.

**Figure 2:**
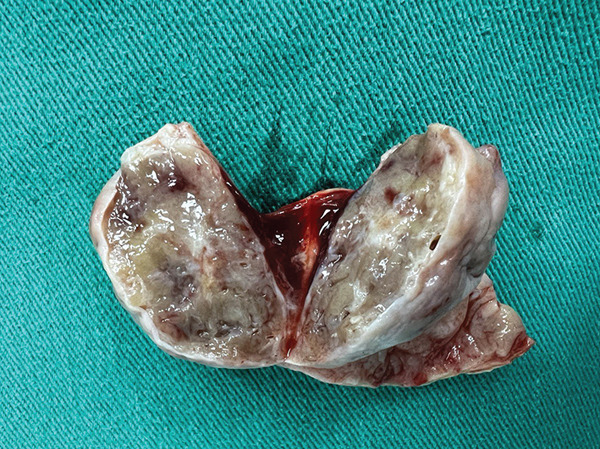
Gross macroscopic appearance of the resected mass.

Macroscopic inspection of the resected mass showed a well‐delineated brown‐colored rubbery mass attached to a segment of the aortic wall (Figure 2). Histological study of the resected mass revealed cuboidal to rounded cell proliferation with normochromic nuclei, forming a near‐alveolar pattern (Figure 3). Immunohistochemistry confirmed positive staining for chromogranin and synaptophysin, negative cytoplasmic keratin, peripheral S100 positivity, and a Ki‐67 proliferative index of < 2%. These findings confirmed the diagnosis of a neuroendocrine paraganglioma (Figure 4).

Figure 3Hematoxylin and eosin stained sections. (a, b) Proliferation within the tunica intima consisting of nests and cords of monotonous cells within a highly vascularized stroma (×40). (c) Distinct nests with fibrovascular septa, displaying a characteristic Zellballen pattern. Tumor cells were polygonal/round, with granular, amphophilic to basophilic cytoplasm. The nuclei exhibited pleomorphism, with occasional large hyperchromatic nuclei. No significant mitotic activity was observed (×200).

**Figure:**
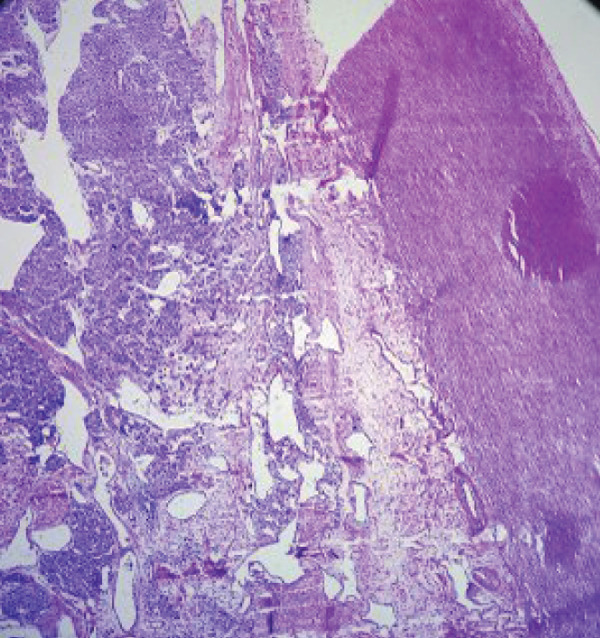
(a)

**Figure:**
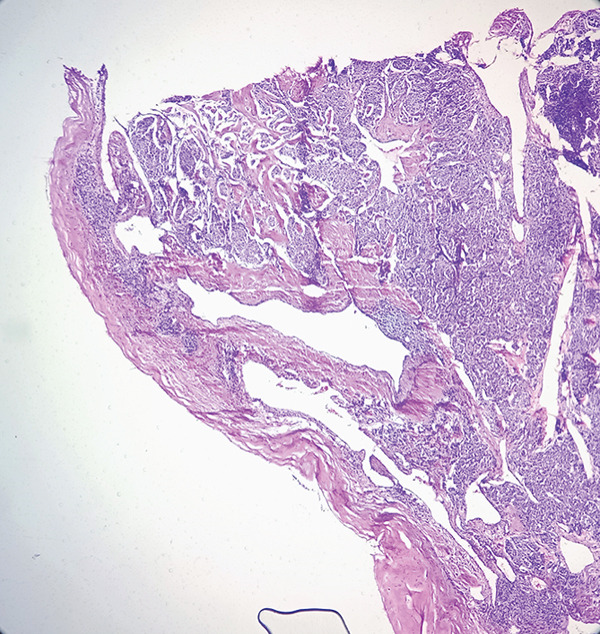
(b)

**Figure:**
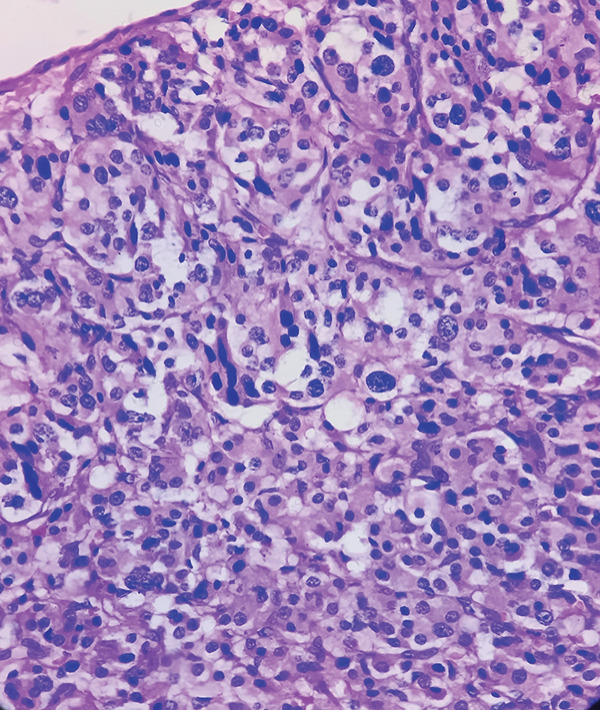
(c)

Figure 4Immunohistochemical staining (×100). (a) S100 accentuates the Zellballen pattern by staining sustentacular cells. (b) Synaptophysin shows distinct positivity in tumor cells. (c) Chromogranin demonstrates strong positivity in tumor cells. (d) Low Ki‐67 proliferative index (< 2%).

**Figure:**
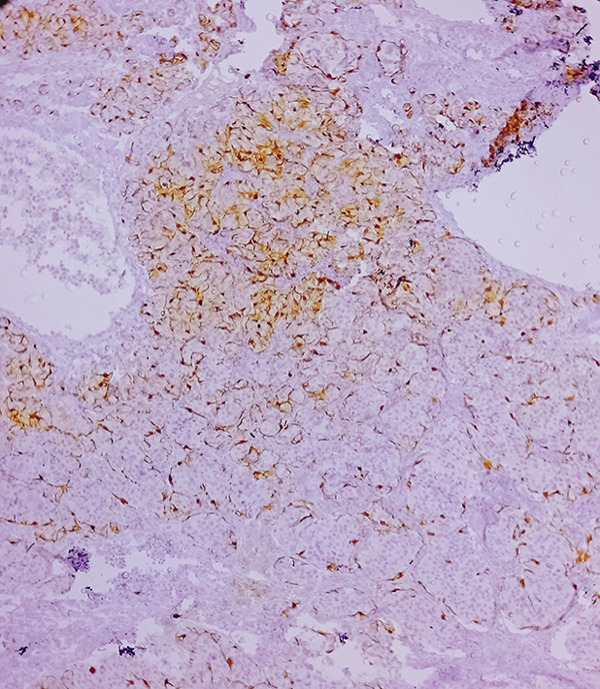
(a)

**Figure:**
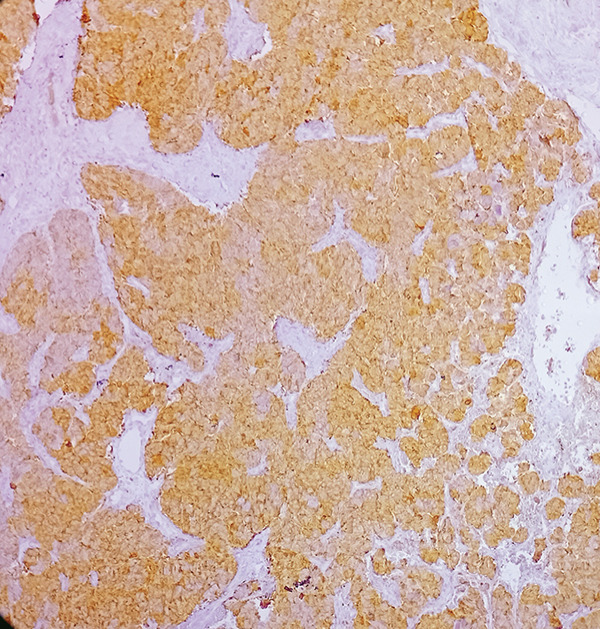
(b)

**Figure:**
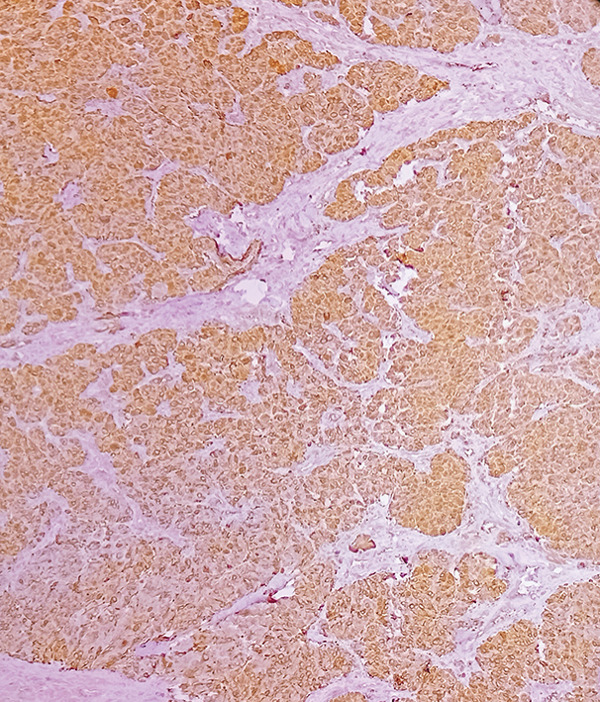
(c)

**Figure:**
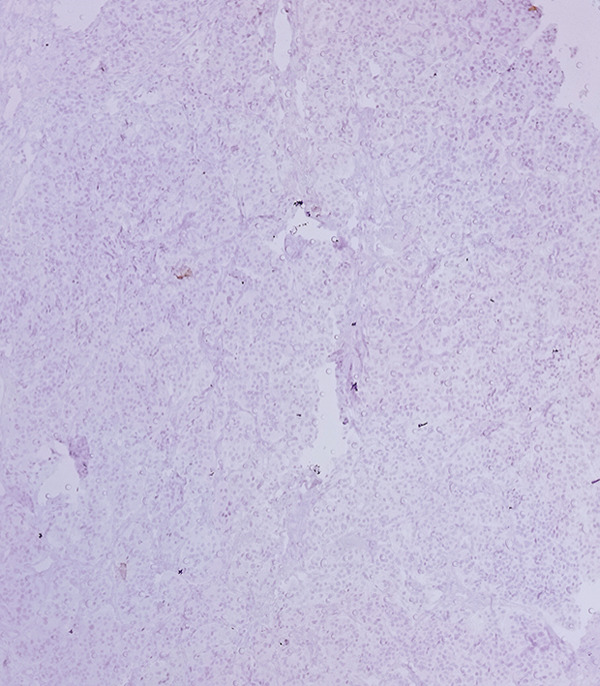
(d)

**Figure 5:**
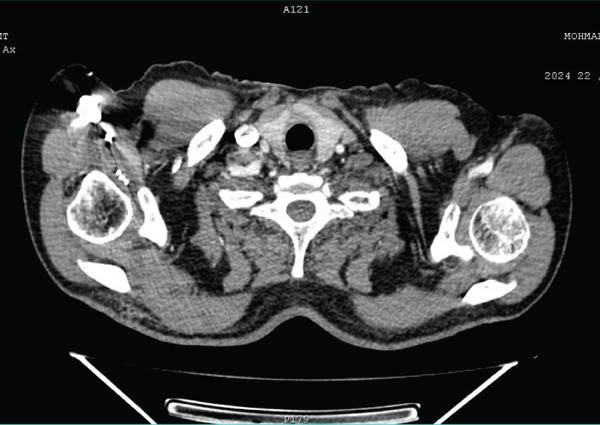
A right carotid body tumor with clear and regular edges and strong homogeneous contrast enhancement.

A computed tomographic scan of the neck–chest–abdomen and pelvis was performed 6 months postoperatively to rule out the presence of multiple neuroendocrine tumors. The right carotid body tumor was noted to have clear and regular edges with strong homogeneous contrast enhancement (Figure 5). The neck–chest–abdomen and pelvis were otherwise normal. Twelve months postoperatively, the patient remains asymptomatic and refuses to undergo any further imaging or hormonal investigation, or surgical excision of the carotid body tumor.

## 3. Discussion

Mediastinal paragangliomas are rare in clinical practice. They arise from ganglia associated with the aorta, pulmonary arteries, or coronary arteries [1], and account for 1%–2% of all paragangliomas and less than 0.3% of mediastinal masses. They typically occur in patients aged 40–50 years regardless of gender [4]. Mediastinal paragangliomas may also be part of a multiple tumor complex, with the carotid body as a frequent adjunct location [4, 5]. This complex has been related to mutations in the B subunit of the SDH hereditary susceptibility genes. Therefore, it has been proposed that all paraganglioma patients, as well as the immediate relatives of patients with SDH gene abnormalities, should undergo genetic testing [4].

Intrapericardial paragangliomas may be accompanied by excessive secretion of catecholamines and their metabolites, causing paroxysmal or persistent hypertension, hyperhidrosis, palpitations, and headache [2]. On the other hand, nonfunctioning mediastinal paragangliomas, which account for approximately 50%–80% of cases [4], may remain asymptomatic until they are uncovered inadvertently during unrelated investigations or interventions [1]. As they grow, nonfunctioning mediastinal tumors often cause symptoms due to tumor compression of surrounding tissues, including cough, dyspnea, and back pain [4].

Anterior mediastinal paragangliomas involving the heart or great vessels may pose a significant surgical challenge. Complete surgical resection is always advocated, and mortality rates after surgical resection are reasonable but may require complex resection and reconstructions, including aortic root replacement or cardiac auto‐transplantation [6]. Long‐term survival rates following surgery are favorable, and local recurrence is very rare [3]. Importantly, the extensive vascularization of these tumors and proximity to critical surrounding structures can lead to life‐threatening bleeding if not controlled. Meticulous preoperative planning, including cardiac magnetic resonance imaging, coronary angiography, and if required, prophylactic embolization, is usually recommended [1, 6]. This should preferably be carried out by a multidisciplinary team [3].

Our patient presented with symptoms of unstable angina but showed no signs for the presence of the tumor. It is arguable that preoperative identification of carotid body tumor should have prompted performing CT scan to rule out presence of multiple tumors, despite the fact that the possibility of simultaneous occurrence of a carotid body tumor and mediastinal paraganglioma remains exceedingly rare [1, 7]. It is now our policy to conduct full‐body CT screening upon discovering a carotid body tumor to rule out the presence of additional tumors and to ensure satisfactory preoperative patient preparation.

In conclusion, this report presents a very rare case where a carotid body tumor was accompanied by the presence of a large nonfunctioning epiaortic paraganglioma. Even though multiple paragangliomas in different locations in the same patient are highly uncommon, we now believe that preoperative discovery of an extrathoracic neuroendocrine tumor should prompt full screening for the presence of multiple tumors, and that this is likely to be associated with improved outcomes.

## Author Contributions

Batoul Abbas, Aya Alhelou, Eman Darrh, and Batool Marwa: acquisition of data, drafting the manuscript, and revising it critically. Albaraa Bara: drafting the manuscript and revising it critically. Mohammad Bashar Izzat: performing surgery, conception, and revising the manuscript critically.

## Funding

No funding was received for this manuscript.

## Disclosure

All authors gave final approval for the version to be published.

## Ethics Statement

All procedures performed in this study were in accordance with the ethical standards of the Damascus University Research Ethics Committee and with the Declaration of Helsinki 1964 and its later amendments.

## Consent

Written informed consent was obtained from the patient for publication of this case report and accompanying images. Ethics committee approval was not applicable.

## Conflicts of Interest

The authors declare no conflicts of interest.

## Data Availability

Data sharing is not applicable to this article as no datasets were generated or analyzed during the current study.

## References

[1] Eto R. , Kawano H. , Horie I. , Kaneko K. , Honda T. , Abe K. , Koga S. , Ikeda S. , and Maemura K. , Paraganglioma of the Carotid Body and Intrapericardium, Journal of Cardiology Cases. (2019) 21, no. 2, 63–66, 10.1016/j.jccase.2019.10.001.PMID. 32042357 PMC6997326

[2] Zhang J. , Cao L. , Yan L. , Jin C. , and Zhang D. , A Young Patient With Heart Failure Was Diagnosed With Extra-Adrenal Paraganglioma: A Case Report, BMC Cardiovascular Disorders. (2022) 22, no. 1, 10.1186/s12872-022-03026-5.PMID. PMC9801580 36581844

[3] Chan E. Y. , Ali A. , Umana J. P. , Nguyen D. T. , Hamilton D. J. , Graviss E. A. , Ravi V. , and Mac Gillivray T. E. , Management of Primary Cardiac Paraganglioma, Journal of Thoracic and Cardiovascular Surgery. (2022) 164, no. 1, 158–166.e1, 10.1016/j.jtcvs.2020.09.100. 33148444

[4] Xu S. , Hu G. , Du J. , Ma L. , Zou L. , and Li Q. , Middle Mediastinal Paraganglioma: A Case Report and Review of the Literature, Medicine (Baltimore). (2023) 102, no. 47, e36327, 10.1097/MD.0000000000036327.PMID. 38013330 PMC10681380

[5] Martucci V. L. , Emaminia A. , del Rivero J. , Lechan R. M. , Magoon B. T. , Galia A. , Fojo T. , Leung S. , Lorusso R. , Jimenez C. , Shulkin B. L. , Audibert J. L. , Adams K. T. , Rosing D. R. , Vaidya A. , Dluhy R. G. , Horvath K. A. , and Pacak K. , Succinate Dehydrogenase Gene Mutations in Cardiac Paragangliomas, American Journal of Cardiology. (2015) 115, no. 12, 1753–1759, 10.1016/j.amjcard.2015.03.020, 2-s2.0-84930181989, 25896150. 25896150 PMC4450109

[6] Yendamuri S. , Elfar M. , Walkes J. C. , and Reardon M. J. , Aortic Paraganglioma Requiring Resection and Replacement of the Aortic Root, Interactive Cardiovascular and Thoracic Surgery. (2007) 6, no. 6, 830–831, 10.1510/icvts.2007.161737, 2-s2.0-37349073352, 17901107. 17901107

[7] Kalekar T. , Rangankar V. , Ayapaneni D. R. , Chanabasanavar V. , and Dhirawani S. , Multiple Paragangliomas in the Carotid Body, Adrenal and Extra-Adrenal Retroperitoneal Locations, Cureus. (2021) 13, no. 9, e18258, 10.7759/cureus.18258, 34722044. 34722044 PMC8544904

